# The Role of Fathers in the Intergenerational Transmission of Obesity

**DOI:** 10.1007/s13679-026-00720-9

**Published:** 2026-05-26

**Authors:** Matthew J. Landry, John James Parker

**Affiliations:** 1 Department of Population Health and Disease Prevention, Joe C. Wen School of Population & Public Health, University of California, Irvine, 856 Health Sciences Rd. Suite 3800, Irvine, CA 92617 USA; 2 Center for Global Cardiometabolic Health and Nutrition, University of California, Irvine, Irvine, CA USA; 3 Family and Child Health Innovations Program, Smith Child Health Outcomes, Research and Evaluation Center, Ann & Robert H. Lurie Children’s Hospital of Chicago, Chicago, IL USA; 4 Departments of Medicine and Pediatrics, Northwestern University Feinberg School of Medicine, Chicago, IL USA

**Keywords:** Paternal, Paternal Origins of Health and Disease, Fatherhood, Obesity, Intergenerational, Child Obesity

## Abstract

**Purpose of Review:**

This narrative review aims to synthesize recent evidence demonstrating that paternal influences can significantly affect child obesity risk through biological, psychosocial, and behavioral pathways and to highlight implications for clinical practice, public health, and research.

**Recent Findings:**

Paternal obesity influences offspring metabolic health through three primary mechanisms. Biologically, paternal obesity alters sperm epigenetic signatures, which are transmissible to offspring and modifiable through preconception lifestyle interventions. Behaviorally, fathers’ dietary quality, physical activity, feeding practices, and parenting styles directly shape children’s eating patterns and activity levels, with the transition to fatherhood representing a critical period when paternal weight gain and lifestyle changes commonly occur. Socioecologically, social determinants of health including food insecurity, neighborhood characteristics, and paternal mental health create shared family-level exposures that simultaneously influence paternal physiology, parenting behaviors, and childhood adversity, compounding obesity risk across generations.

**Summary:**

Fathers play a pivotal role in shaping child obesity risk from preconception through childhood. Advancing obesity prevention requires expanding preconception counseling to include fathers, implementing father-inclusive perinatal education and obesity prevention programs, adopting workplace policies that support paternal engagement, and prioritizing research that elucidates paternal contributions to intergenerational obesity transmission.

## Introduction

Obesity remains a major public health challenge, with prevalence rising in children, adolescents, and adults [1]. If the current pattern continues, more than 250 million people living in the United States will have overweight or obesity by 2050 [2]. Parental obesity is a strong predictor of pediatric obesity and the risk is highest when both parents are affected [3, 4]. The persistence of obesity across generations has prompted increased recognition that obesity is not merely the result of individual lifestyle choices, but a chronic disease that emerges from complex interactions between genetic, biological, behavioral, and environmental factors that can span multiple generations [5]. 

The Developmental Origins of Health and Disease (DOHaD) paradigm posits that environmental exposures during critical windows of development, particularly the periconceptional, fetal, and early postnatal periods can program long-term health outcomes, including obesity and metabolic disease, in offspring [6, 7]. Historically, DOHaD research has focused on maternal influences, such as nutrition, obesity, and metabolic status, which shape the intrauterine environment and fetal development through mechanisms including epigenetic modification, placental function, and metabolic programming [7, 8]. More recent advances have expanded this framework to include the Paternal Origins of Health and Disease (POHaD), recognizing that paternal health and exposures prior to conception, such as obesity, diet, age, and environmental toxins, can also program offspring risk for obesity and related disorders [9–12]. 

Both DOHaD and POHaD frameworks are now recognized as complementary and essential for understanding intergenerational transmission of obesity risk [11, 13]. While maternal factors remain critical, paternal contributions are increasingly shown to have substantial and sometimes unique effects on offspring metabolic health outcomes [12, 14, 15]. Additionally, emerging evidence demonstrates that paternal influences on offspring health extend from the preconception period through well beyond the perinatal window, with long-term implications for child obesity risk [16, 17]. 

This review aims to synthesize recent evidence demonstrating that paternal influences can significantly influence child obesity risk through both biological, psychosocial, and behavioral pathways. Secondarily, this review seeks to highlight clinical, public health, and research implications of more fully incorporating fathers into efforts to prevent intergenerational obesity.

### Pathways Linking Paternal and Offspring Obesity Risk

**Fig. 1:**
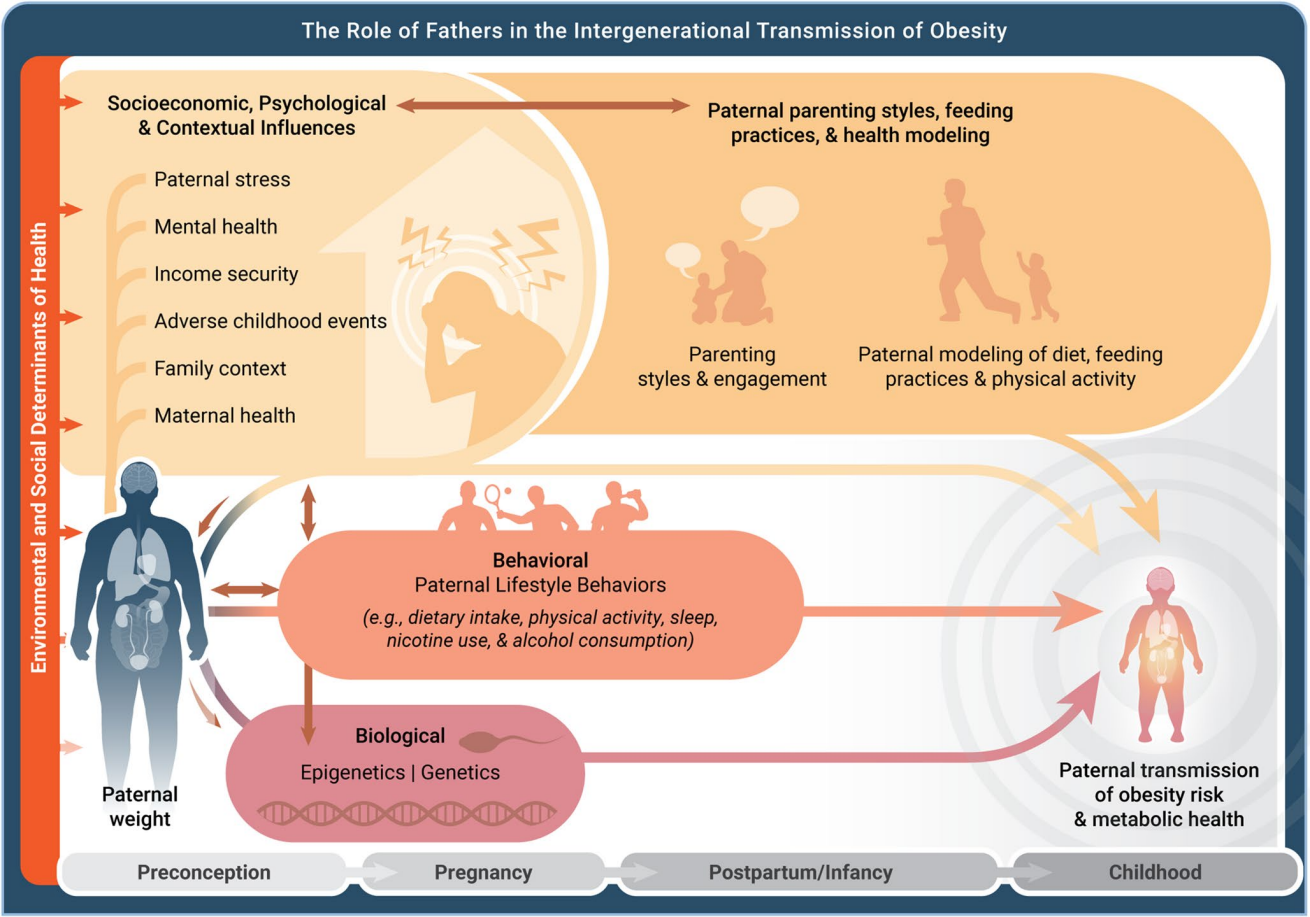
Conceptual model of mechanisms through which paternal factors contribute to the intergenerational transmission of child obesity risk

Grounded in the DOHaD framework and the emerging POHaD paradigm, Fig. 1 provides a conceptual model delineating mechanisms through which paternal factors contribute to the intergenerational transmission of obesity risk and metabolic health outcomes from preconception through childhood. Biological pathways operate through epigenetic modifications and genetic inheritance, whereby paternal obesity-related genetic and epigenetic signatures are transmitted to offspring, potentially programming metabolic risk across generations. Behavioral pathways encompass paternal lifestyle behaviors, including dietary patterns, physical activity levels, and other health-related practices that shape the home environment and establish behavioral norms for children. Socioecological, psychological, and contextual influences represent upstream determinants, including paternal stress, mental health status, income security, adverse childhood experiences, family structure, and maternal health, which collectively influence both paternal behaviors and child developmental contexts. Paternal parenting styles, feeding practices, and health modeling serve as proximal mediators through which fathers’ engagement patterns, parenting approaches, and role modeling of dietary and physical activity behaviors directly influence children’s health trajectories. Underlying each of these pathways is the recognition that paternal influences are best understood within a dyadic framework, in which fathers, mothers and other caregivers often function as an interconnected system whose combined behaviors, relationship quality, and mutual support collectively shape the environments in which children grow and develop [18]. Each of the previously described pathways operate within overarching environmental and social determinants of health that create bidirectional influences between individual-level factors and broader structural conditions, ultimately shaping childhood obesity risk and metabolic health across critical developmental periods spanning preconception, pregnancy, postnatal/infancy, and childhood.

### Paternal Biological Contributions: Genetic, Epigenetic, and Molecular Mechanisms

A central mechanism underlying the intergenerational transmission of obesity is the genetic and molecular material that fathers contribute at conception [19]. Obesity is approximately 40–70% heritable, with genetic susceptibility shaped by hundreds of common variants interacting with environmental exposures [20]. Large-scale genome-wide analyses have identified paternal-transmitted variants in genes such as FTO, MC4R, TMEM18, and LEPR, among many others, that contribute to adiposity and metabolic regulation [21, 22]. For example, inheriting one risk allele at FTO is associated with a 20–30% higher BMI, whereas inheriting two alleles increases obesity risk by 60–70% [23, 24]. 

Unlike maternal oocytes, paternal germ cells undergo continuous renewal across the life course. Spermatogenesis begins approximately 74 days prior to ejaculation, and the final stages remain highly sensitive to metabolic, endocrine, inflammatory, and environmental conditions in the 3–6 months before conception [25, 26]. As a result, paternal physiology, including obesity, diet quality, stress, toxin exposure, sleep, and physical activity, directly influences sperm quality and the molecular cargo packaged within sperm [14, 27–29]. For example, compared with men in the normal-weight range, men with obesity have 20–40% lower sperm concentration, reduced motility, and higher rates of sperm DNA fragmentation, reflecting the detrimental effects of metabolic dysregulation on reproductive function [30–32]. For men with obesity, these alterations to sperm production and quality increase the risk of infertility by 30–66% and increase the odds of pregnancy loss independent of maternal factors [33, 34]. 

Several biological mechanisms link paternal obesity to impaired sperm function and to offspring metabolic programming. First, obesity leads to endocrine disruption: circulating testosterone levels are lower in men with obesity due to hypothalamic–pituitary–gonadal axis suppression and increased aromatization [32, 33, 35]. Second, obesity stimulates both systemic and testicular inflammation, which impairs Sertoli cell function and induces DNA fragmentation and sperm mitochondrial dysfunction [14, 36]. Third, obesity drives epigenetic alterations in sperm. Sperm DNA methylation, histone retention patterns, and small non-coding RNA profiles are highly responsive to metabolic conditions and have the capacity to regulate gene expression in early embryogenesis [37]. Obesity is associated with differential methylation in genes regulating appetite, insulin signaling, and adipocyte biology [38]. DNA methylation patterns can activate or silence specific genes and may influence which genetic sequences are selected or prioritized during spermatogenesis. Animal studies show that paternal high-fat diet reprograms the sperm methylome and induces adiposity, impaired glucose tolerance, and pancreatic dysfunction in offspring [39]. 

Human studies further demonstrate that these epigenetic signals are dynamic and reversible. Weight loss interventions, including both behavioral and surgical approaches, modify sperm DNA methylation patterns and improve sperm quality and function. For example, paternal bariatric surgery leads to rapid methylation changes in metabolic genes [40], and lifestyle-based interventions yield substantial improvements in motility, morphology, and DNA integrity beyond what can be attributed to weight loss alone [41]. Together, these biological pathways underscore the preconception period as a critical window for influencing paternal epigenome contributions to offspring health. However, human mechanistic research is limited and primarily observational, with few studies fully elucidating the biological pathways by which paternal health at conception influences future child obesity risk [17]. 

### Lifestyle and Health Behaviors of Fathers

The transition to fatherhood among men represents a major, discrete life event in which substantial changes to lifestyle and health behaviors may occur [42]. Some men may be motivated to improve their health during the transition to fatherhood, while others are challenged to choose a healthy lifestyle and engage in health promoting behaviors as they experience disruption to their typical routines [43]. A father’s lifestyle and health behaviors from preconception through his child’s early childhood can have impacts on both his health and the health of his child.

The mechanisms linking paternal lifestyle behaviors to child obesity risk operate through interconnected biological and social pathways. Behavioral modeling represents a primary social mechanism, with children observing and imitating paternal eating patterns, activity levels, and health-related attitudes [44]. Paternal parenting style also significantly moderates the relationship between father behaviors and child outcomes [45]. Research demonstrates that greater father involvement in childcare is associated with healthier child dietary patterns and lower obesity risk, independent of socioeconomic factors [46, 47]. Disengaged parenting is associated with detrimental impacts on children’s lifestyle behaviors [48, 49]. Research additionally suggests that when both parents model healthy behaviors and employ consistent parenting approaches, effects on child obesity risk are strengthened [44, 50, 51]. 

As fathers adjust to new responsibilities and time constraints during the transition from preconception through pregnancy and into the postpartum and early childhood periods, they commonly experience modest but significant increases in weight and BMI. Longitudinal studies show that paternal BMI, body fat percentage, and waist circumference tend to rise from early pregnancy to at least 6 months postpartum, reflecting unfavorable changes in body composition [52–55]. For example, one prospective study found that fathers’ BMI increased by approximately 0.22 kg/m² from prebirth to 5–6 months postpartum, with these changes persisting at 12 months [55]. Another study confirmed increases in BMI, body fat, and waist circumference from pregnancy to 6 months postpartum [52]. These weight gain patterns may stem from fathers not engaging in healthy behaviors during this transition period. Lifestyle factors such as decreased physical activity, increased intake of unhealthy foods, disrupted sleep, and heightened stress can contribute to unfavorable weight trajectories in new fathers [43]. Multiple large cohort studies demonstrate that children of fathers with overweight or obesity have higher odds of being overweight or obese themselves, independent of maternal weight status [56–58]. Given that children of fathers with obesity have increase odds of obesity independent of maternal factors, further characterizing and addressing the weight gain associated with fatherhood may help prevent the intergenerational transmission of obesity.

Among lifestyle and health behaviors, a father’s diet may be one of the strongest factors shaping his child’s obesity risk, from preconception through the entirety of the child’s development. Emerging research in paternal nutritional programming suggests that fathers’ dietary patterns before conception may influence offspring metabolic health through epigenetic modifications in sperm [16, 59]. Diets rich in fruits, vegetables, whole grains, nuts, seafood, and low-fat dairy are linked to higher sperm concentration, motility, morphology, and total sperm count, independent of age and BMI [60, 61]. Conversely, high consumption of red and processed meats, saturated fats, sugar-sweetened beverages, and ultra-processed foods is associated with poorer sperm quality, including lower motility, abnormal morphology, and increased DNA fragmentation [62]. 

During the pregnancy period, fathers’ dietary behaviors contribute to shaping the household food environment [63]. Research indicates that paternal food preferences and purchasing decisions influence the nutritional quality of foods available in the home, indirectly affecting maternal diet quality during pregnancy [64, 65]. Partners of pregnant women often share similar dietary patterns, suggesting that paternal nutrition during this period may have cascading effects on fetal nutrition through maternal dietary intake [66]. Mothers are typically considered the primary feeder in a child’s life, beginning in infancy with breastfeeding. A fathers’ attitudes toward parenthood and positive attitudes toward breastfeeding are associated with increased likelihood of breastfeeding initiation and continuation, even though they are not directly feeding the infant [67]. Breastfeeding is associated with a lower risk of childhood overweight and obesity, with a dose-response relationship observed in many studies [68]. 

As children transition to solid foods and through early childhood, fathers’ direct involvement in feeding may increase, and their influence becomes more pronounced through both modeling and feeding practices. Fathers’ own dietary intake is predictive of their children’s intake [44, 69]. Research has identified distinct paternal feeding styles that influence child eating behaviors and weight status [70]. Fathers tend to employ more permissive or uninvolved feeding approaches compared to mothers, including pressure to eat, restriction, and using food as a reward [71]. However, fathers who engage actively in meal preparation and family dining create structured eating environments associated with healthier child dietary patterns and reduced obesity risk [72]. Positive modeling, monitoring, and shared family meals are associated with better diet quality and healthier food choices in children [44, 73]. 

Pregnancy also represents a time when prospective fathers may reduce their own physical activity as they prepare for parenthood, potentially establishing sedentary patterns that persist. Paternal physical activity levels show variation during the postpartum period, with research generally documenting decreases in father physical activity following childbirth [54, 74, 75]. Fathers experience significant sleep disruptions during the postpartum period, though typically less severe than maternal sleep disturbance [55, 68]. These sleep challenges can affect fathers’ energy levels for physical activity and their ability to maintain healthy lifestyle routines. Evidence indicates that fathers play a unique role in promoting child physical activity during early childhood [76]. Studies demonstrate positive associations between father-child physical activity co-participation and children’s moderate-to-vigorous physical activity levels [77]. Fathers who model active lifestyles and create opportunities for family physical activity have children with higher overall activity levels and reduced screen time [78]. Conversely, paternal sedentary behaviors, particularly screen-based activities, influence family screen time norms and can be predictive of similar sedentary behaviors in children [79]. Notably, sedentary behavior is not simply the absence of physical activity but a distinct risk factor with independent effects on metabolic health, even among those who are otherwise physically active [80]. Large cohort and cross-sectional studies consistently demonstrate a dose-response relationship between child screen time and increased BMI percentile or risk of overweight/obesity, even after accounting for physical activity levels [81, 82]. Beyond their own activity behaviors, fathers may also influence offspring health indirectly through their effect on maternal physical activity during pregnancy. Perceived partner support has been positively associated with maternal exercise participation and adherence during pregnancy [83]. 

A father’s substance use of tobacco and alcohol can have significant consequences for child health. Research demonstrates that paternal smoking is associated with DNA damage and epigenetic modifications in sperm, including altered methylation patterns in genes that regulate growth and metabolism [10, 84]. Epidemiological studies have also linked paternal preconception smoking to increased offspring obesity risk, with some evidence suggesting dose-response relationships between smoking intensity and child BMI [85, 86]. Paternal alcohol consumption before conception similarly impacts sperm quality and epigenetic programming [87]. Human studies have found associations between paternal alcohol use and offspring neurodevelopmental outcomes, though relationships with obesity remain less well-characterized [88]. Beyond these biological mechanisms operating through preconception exposures, paternal substance use during a child’s upbringing may also influence offspring health behaviors through social modeling, representing a distinct developmental pathway [89]. 

### Socioeconomic, Psychological, and Contextual Influences

Paternal contributions to the intergenerational transmission of obesity are deeply shaped by the socioeconomic, psychological, and contextual environments in which fathers live. Social determinants of health (SDOH), including income, education, food access, neighborhood safety, exposure to discrimination, and structural inequities, affect fathers’ health behaviors, stress physiology, mental health, and obesity risk [90, 91]. These paternal factors, in turn, influence the developmental environments to which children are exposed, contributing to obesity risk across generations.

There is consistent evidence that lower socioeconomic status is associated with substantially higher obesity risk in adults and children [92]. Food insecurity, experienced by millions of families globally, is associated with higher consumption of energy-dense foods, disrupted eating patterns, and increased odds of obesity [93, 94]. Neighborhood characteristics also play a key role; systematic reviews indicate that living in neighborhoods with limited access to recreational spaces, poor walkability, and high crime rates is associated with higher BMI, reduced physical activity, and increased cardiometabolic risk [95]. These contexts constrain opportunities for health-promoting behaviors for fathers and family units more broadly.

Psychological factors serve as another key pathway linking socioeconomic context to paternal obesity and subsequent child health. A meta-analysis demonstrated strong bidirectional associations between depression and obesity, with individuals experiencing depression having a 40% increased risk of developing obesity and individuals with obesity having a 57% increased risk of depression [96]. Fathers living in disadvantaged contexts have higher rates of psychological distress, stress, and depressive symptoms, which can alter dietary habits, decrease physical activity, disrupt sleep, and elevate inflammation [97]. Importantly, paternal mental health also affects family functioning [98, 99]. Paternal depression increases the likelihood of adverse parenting behaviors, lower engagement, and decreased emotional availability, each of which has been associated with unhealthy child eating behaviors, disordered sleep patterns, and higher obesity risk [98–100]. 

The interaction between paternal and maternal mental health further amplifies risk within families. Paternal depression is associated with a significantly increased risk of maternal depression during both the prenatal and postpartum periods [101]. Furthermore, paternal depression is associated with their family’s healthcare utilization; partners of depressed fathers have reduced attendance at prenatal care and postpartum visits and their children have lower attendance at well-child appointments [99, 102]. Reduced engagement with preventive health care decreases opportunities for early identification of feeding challenges, growth concerns, or social stressors that influence child obesity trajectories [103]. 

This compounding effect of parental mental health on family functioning becomes particularly concerning when examining the broader context of childhood adversity. Children living with a parent with depression endure at least one Adverse Childhood Experiences (ACE), and many will have multiple ACEs [104, 105]. ACEs are potentially traumatic or stressful events that occur during childhood and have lasting impact of health and wellbeing and risk for obesity across the life course [105]. Individuals who experience an ACE have an increased risk of obesity and there is a dose-dependent relationship between number of ACEs and obesity risk [106]. Mechanistically, ACEs influence stress response systems, emotional regulation, sleep, and health behaviors, contributing to higher abdominal adiposity and long-term cardiometabolic dysregulation [107]. Importantly, the same structural conditions that increase obesity risk in fathers, poverty, discrimination, food insecurity, neighborhood disinvestment, also increase the likelihood that children will experience ACEs [107]. Thus, socioeconomic adversity and psychological stressors create a shared family-level exposure that simultaneously influences paternal physiology, parenting behaviors, and child developmental environments [91, 108]. 

## Conclusion

### Clinical and Public Health Implications

Fathers play a pivotal role in shaping child obesity risk, through both direct and indirect influences from preconception through postnatal periods and into childhood. Recognizing the critical influence of paternal health on offspring outcomes necessitates expanding preconception counseling beyond its traditional maternal focus to actively engage prospective fathers [10, 109]. Prospective fathers who participate in prenatal visits are more likely to adopt healthy behaviors, support maternal wellness, and engage in early parenting, which can reduce childhood obesity risk and enhance family functioning. Despite these benefits, paternal engagement during prenatal visits remains suboptimal, with many fathers reporting limited inclusion and unmet informational needs [110]. To address this gap, healthcare systems can implement father-inclusive practices that normalize and encourage paternal involvement. This includes directly inviting fathers to prenatal appointments, providing them with targeted health information and resources, and training healthcare providers to engage fathers as active partners in family health [111]. Clinicians can additionally counsel fathers on the importance of their own health and lifestyle behaviors, including weight management, physical activity, nutrition, and stress reduction not only for their personal wellbeing but also for their potential impact on their children’s health trajectories [112]. 

Available evidence suggests that father-inclusive programs are associated with enhanced partner relationship quality, better father’s mental health, and more supportive paternal behaviors [113]. Fathers are additionally often underrepresented in child obesity prevention programs [50, 114]. Education programs should be redesigned to explicitly recruit, engage, and retain fathers through culturally responsive messaging, convenient scheduling, and content that addresses fathers’ specific concerns and interests [115, 116]. Programs can additionally acknowledge the diversity of family structures and recognize that father figures, including stepfathers, adoptive fathers, and other male caregivers, all play important roles in shaping children’s health [117]. 

Structural barriers in the workplace significantly limit fathers’ ability to engage in health-promoting behaviors and participate actively in their children’s lives during critical developmental periods. The adoption of workplace policies that support paternal health and family needs, including adequate paid paternal leave and flexible work arrangements, is critical for enabling fathers to be present and engaged during the perinatal period and early childhood. These policies and practices are often associated with improved mental health and family wellbeing [118–120]. In the US, current policies often fall short due to entrenched gender norms and insufficient support. There is currently no universal paid family and medical leave. The Family and Medical Leave Act (FMLA) provides up to 12 weeks of unpaid, job-protected leave, but more than half of employees are ineligible, and most eligible workers cannot afford to take unpaid leave [121]. This creates significant disparities in access to parental leave, disproportionately affecting low-income families and perpetuating health inequities. In stark contrast, almost all high income countries offer extensive paid parental leave, with high wage replacement rates and specific quotas for fathers to incentivize paternal involvement [119, 122]. Nordic countries (e.g., Sweden, Norway, Iceland) are often considered exemplary models for parental leave policy due to their extensive paid leave provisions, high wage replacement rates, and father-specific leave incentives designed to promote paternal involvement in caregiving. Compared with the limited and unpaid leave structure in the US, these international models demonstrate how policy environments can not only support gender equity in caregiving but also create opportunities for fathers to establish healthy feeding practices, physical activity routines, and emotional bonding with their children during formative developmental periods. Policy advocacy can prioritize policies and practices that create environments where fathers can fulfill their important role in obesity prevention and child health promotion.

The evidence synthesized in this review reflects a research landscape that has overwhelmingly studied two-parent, heterosexual households. In same-sex parent families, particularly those headed by two fathers, biological pathways remain operative through the biological father, whether a co-parent or sperm donor. The behavioral and environmental pathways discussed in this review may manifest differently; however, no identified study has specifically measured child obesity or BMI as an outcome variable in same-sex parent families. The consistent finding that family processes (e.g., relationship quality, parenting competence, socioeconomic stability) matter more than family structure for child outcomes suggests that obesity risk in these families is likely driven by the same modifiable factors as in any family [123, 124]. Similarly, father-headed single-parent households are also historically underrepresented in research; however, it is expected that they could introduce a structurally different set of social, behavioral, and environmental conditions that likely modulate child obesity risk. Obesity prevalence is elevated in single-parent households broadly, with children showing higher BMI z-scores both cross-sectionally and longitudinally compared to two-parent families [125, 126]. However, one large U.S. study found children in single-father families did as well as or better than children with two biological parents on physical health measures, likely reflecting selection effects [127]. Across family types, social support structures, household resource availability, and neighborhood-level environmental exposures likely serve as critical moderating variables.

### Future Research Directions

As gender norms evolve and paternal involvement in caregiving shifts, the scientific community must ensure research frameworks reflect fathers’ critical and equal role in shaping offspring health trajectories. There is a lack of research in three areas: paternal health from preconception to postpartum, mechanistic studies of paternal influence on child obesity risk, and interventional studies that including fathers in child obesity prevention.

Men’s health during the transition to fatherhood is understudied compared to maternal and child health primarily due to historical, cultural, and practical factors. Historically, research and public health initiatives have focused on mothers, based on the assumption that maternal health has the most direct impact on pregnancy and child outcomes [13, 128]. Fathers were not recognized as being at risk for perinatal mental illnesses or as relevant to maternal and infant health outcomes, leading to their exclusion from surveillance systems, clinical guidelines, and intervention studies [129, 130]. Practical barriers have further limited paternal research. Fathers are less likely to be engaged in perinatal research, often because they are not directly approached or included in study designs, and there are fewer routine healthcare touchpoints for men during the perinatal period [129]. Additionally, future research on child obesity risk should prioritize the inclusion of diverse family structures (e.g., same-sex parent families and father-headed single-parent households) and adopt more flexible conceptualizations of parental roles.

While animal studies have elucidated many pathways, human data remain limited, especially regarding the persistence and functional impact of epigenetic changes in offspring. Future research can prioritize human studies that investigate potential mechanisms of paternal influence. Integration of biological data with detailed behavioral, environmental, and psychosocial assessments will enable a more comprehensive understanding of how paternal factors interact to influence child obesity risk. For example, the National Institutes of Health Environmental influences on Child Health Outcomes (ECHO) program is cited as a model for collecting comprehensive paternal, maternal, and child data to study these complex interactions [18]. 

Longitudinal research with fathers is crucial for understanding both their own health trajectories and the intergenerational impact on child outcomes. The relative scarcity of studies with repeated outcome measures across childhood limits conclusions about when paternal effects first manifest versus when they simply become detectable given current study design. Prospective cohort studies can capture changes in paternal physical health, mental health, lifestyle behaviors, and psychosocial factors from preconception through the postpartum period and beyond, providing insights into how these changes influence child development, obesity risk, and long-term family wellbeing. Several cohort studies such as the Australian-based Men and Parenting Pathways (MAPP) Study [131], the United Kingdom-based New Dad Study (NEST) [132], the Australian-based Ten to Men Study [133], are addressing this research gap by tracking young men during their transition into fatherhood and subsequently their health, mental wellbeing, and lived experiences throughout early parenthood. Forthcoming work such as the US-based Dad Bod Study will contribute additional information about both acute and longer-term changes in health associated with new paternal roles and responsibilities [42]. Expanding and sustaining such cohorts is essential for building the evidence base needed to develop targeted interventions and inform clinical guidelines that support fathers’ health and optimize their contributions to child health outcomes.

Lastly, there is a critical need for interventional studies that test the efficacy of father-inclusive approaches to childhood obesity prevention [50]. Such studies should employ randomized controlled trial designs to evaluate whether engaging fathers in health promotion programs, beginning in the preconception period and continuing through early childhood, improves family health behaviors and reduces child obesity risk compared to traditional mother-focused interventions. One such example is the US-based First Heroes effectiveness-implementation randomized trial of mother-father-infant triads [134]. Interventions should be theory-driven, culturally tailored, and designed to address known barriers to paternal engagement. Research should also examine which intervention components are most effective, what dosage and timing optimize outcomes, and whether effects differ across diverse populations defined by race, ethnicity, socioeconomic status, and family structure [129, 135]. 

Advancing child obesity prevention requires a paradigm shift to fully integrate fathers into clinical, public health, and research frameworks. This includes developing father-inclusive programs, supportive workplace and healthcare policies, and rigorous research that addresses biological, behavioral, and social determinants.

**Table:** 

Annotated Reference List of Important and Very Important Recent References			
Important	Ref Number	Reference	Annotation
*	[29]	Noor N, Cardenas A, Rifas-Shiman SL, Pan H, Dreyfuss JM, Oken E, et al. Association of periconception paternal body mass index with persistent changes in DNA methylation of offspring in childhood. JAMA Netw Open. 2019;2(12):e1916777-e.	Paternal BMI associated with DNA methylation, birth weight, and childhood BMI z score in offspring.
*	[54]	Versele V, Stas L, Aerenhouts D, Deliens T, Matthys C, Gucciardo L, et al. Dietary intake, physical activity and sedentary behavior and association with BMI during the transition to parenthood: a prospective dyadic study. Front Public Health. 2023;11:1092843.	Fathers experienced unfavorable changes in lifestyle behaviors during the transition to fatherhood.
*	[57]	Rossi A, Chen ZH, Ahmadiankalati M, Campisi SC, Reyna ME, Dempsey K, et al. Determining the interplay of prenatal parental BMI in shaping child BMI trajectories: the CHILD Cohort Study. Int J Obes (Lond). 2025;49(8):1608-15.	Children of fathers with obesity had higher odds of being in the rapid growth BMI trajectory.
*	[69]	Rahill S, Kennedy A, Kearney J. A review of the influence of fathers on children’s eating behaviours and dietary intake. Appetite. 2020;147:104540.	Describes fathers’ influence on child eating behaviors and dietary intake
*	[55]	Lo BK, Kang AW, Haneuse S, Yu X, Ash TV, Redline S, et al. Changes in Fathers’ Body Mass Index, Sleep, and Diet From Prebirth to 12 Months Postbirth: Exploring the Moderating Roles of Parenthood Experience and Coparenting Support. Ann Behav Med. 2021;55(12):1211-9.	Fathers self-reported higher BMI and decreased sleep duration after their child’s birth.
Very Important	Ref Number	Reference	Annotation
**	[37]	Akhatova A, Jones C, Coward K, Yeste M. How do lifestyle and environmental factors influence the sperm epigenome? Effects on sperm fertilising ability, embryo development, and offspring health. Clin Epigenetics 2025;17(1):7. (In eng). DOI: 10.1186/s13148-025-01815-1.	Paternal lifestyle behaviors and exposure to toxic-endocrine-disrupting chemicals linked to transgenerational transmission of increased predisposition to disease
**	[41]	Peel A, Lyons H, Tully CA, Vincent AD, Jesudason D, Wittert G, et al. The effect of obesity interventions on male fertility: a systematic review and meta-analysis. Hum Reprod Update. 2025.	Improvements in semen quality following lifestyle interventions suggest a potential benefit of optimizing nutrition and physical activity.
**	[56]	Chodick G, Simchoni M, Jensen BW, Derazne E, Pinhas-Hamiel O, Landau R, et al. Heritability of Body Mass Index Among Familial Generations. JAMA Netw Open. 2024;7(6):e2419029-e.	Offspring born to parents with obesity during late adolescence had a greater probability of having obesity
**	[70]	Lozano-Casanova M, Gutierrez-Hervas A, Richart-Martinez M, Oliver-Roig A, Sospedra I. Paternal feeding practices and styles: a systematic review. Nutr Rev. 2024;82(6):794–803.	Fathers show a tendency toward an authoritarian feeding style and coercive feeding practices.
**	[98]	Le Bas G, Aarsman SR, Rogers A, Macdonald JA, Misuraca G, Khor S, et al. Paternal Perinatal Depression, Anxiety, and Stress and Child Development: A Systematic Review and Meta-Analysis. JAMA Pediatr. 2025.	Father’s mental state may exert a more direct influence on the developing child development outcomes after birth.

## Data Availability

No datasets were generated or analysed during the current study.
